# Therapeutic approaches in Hirschsprung’s disease: clinical cases

**DOI:** 10.25122/jml-2024-0307

**Published:** 2024-06

**Authors:** Viorel Țandea, Ionuț Daniel Răducan, Oana Neagu, Silviu Constantinoiu

**Affiliations:** 1 Department of Pediatric Surgery, Grigore Alexandrescu Emergency Clinical Hospital for Children, Bucharest, Romania; 2 Department of Plastic and Reconstructive Surgery, Pediatric Surgery, Faculty of Medicine, Carol Davila University of Medicine and Pharmacy, Bucharest, Romania; 3 Department of Pharmaceutical Sciences, Vasile Goldiş Western University of Arad, Arad, Romania; 4 Department of Anatomopathology, Grigore Alexandrescu Emergency Clinical Hospital for Children, Bucharest, Romania; 5 Department of General Surgery, Sf. Maria Clinical Hospital, Bucharest, Romania

**Keywords:** Hirschsprung’s disease, congenital megacolon, clinical cases

## Abstract

Hirschsprung’s disease is a congenital disorder characterized by the absence of ganglion cells in the myenteric and submucosal plexuses of the colon, resulting in impaired peristalsis and functional intestinal obstruction. This condition affects approximately 1 in 5,000 newborns, with a higher prevalence in boys. Although first identified in the 17^th^ century, its connection to chronic constipation was clearly established by Harald Hirschsprung in 1886. Contemporary treatment strategies emphasize early diagnosis, surgical excision of the aganglionic segment, and specialized postoperative care to restore normal colonic function and improve the quality of life for affected individuals. This article reviews current therapeutic strategies, highlighting advanced surgical techniques, diagnostic methods, and postoperative management. Two clinical cases illustrate the impact and treatment of the disease. The first case involves a 5-month-old male with severe malnutrition, dehydration, and acute enterocolitis, who underwent a right-side colostomy and later a Duhamel-type extramucosal sphincter-rectal-myotomy, showing favorable progress. The second case is a 1.5-year-old patient with a history of colostomy and ileostomy, requiring further surgery. Histopathological examinations in both cases revealed the absence of ganglion cells, confirming the diagnosis of Hirschsprung’s disease.

## INTRODUCTION

Hirschsprung’s disease, also known as congenital megacolon, is a congenital condition characterized by the absence of ganglion cells in the myenteric and submucosal plexuses of the colon, resulting in a lack of peristalsis in the affected segment of the intestine. This condition results in a functional intestinal obstruction that can vary in severity depending on the length of the affected colon segment [1]. The incidence of the disease is estimated at 1 in 5,000 newborns, being more common in boys than in girls [2].

This disease was first described in the 17^th^ century, but it was only in 1886 that Danish pediatrician Harald Hirschsprung identified the connection between chronic constipation in children and congenital megacolon [3,4]. Over the years, significant progress has been made in understanding etiopathogenesis and developing treatment strategies, culminating with the introduction of surgical procedures for the excision of the aganglionic segment.

Modern treatment of Hirschsprung’s disease involves a combination of early diagnosis, surgical management, and specialized postoperative care. Although surgical techniques have improved considerably, the primary goal remains to restore intestinal continuity and normal colonic function while reducing the risk of complications and improving patients’ quality of life [5,6].

This communication highlights current therapeutic approaches in Hirschsprung’s disease through a detailed analysis of two clinical cases, emphasizing innovative surgical techniques and postoperative management strategies. We will discuss both the theoretical and practical aspects of the treatment, providing an overview of recent advances and future directions in managing this complex condition.

## MATERIAL AND METHODS

**Case 1:** A 5-month-old male infant presented with significant abdominal distension and was suspected of megadolichocolon. The patient was transferred from a county hospital and diagnosed with grade 3 protein-calorie malnutrition, acute enterocolitis, and severe dehydration syndrome complicated by collapse. The patient had an unfavorable evolution. The family medical history revealed that the mother was 21 and congenitally deaf-mute, and the father was 33 and reported to be in good health. The infant has a 17-year-old half-sister who is reportedly healthy, and his paternal grandmother has a history of diaphragmatic hernia. The personal medical history indicated that this child is the mother’s firstborn, following a partially investigated pregnancy, and was delivered by cesarean section at term.

**Figure 1:**
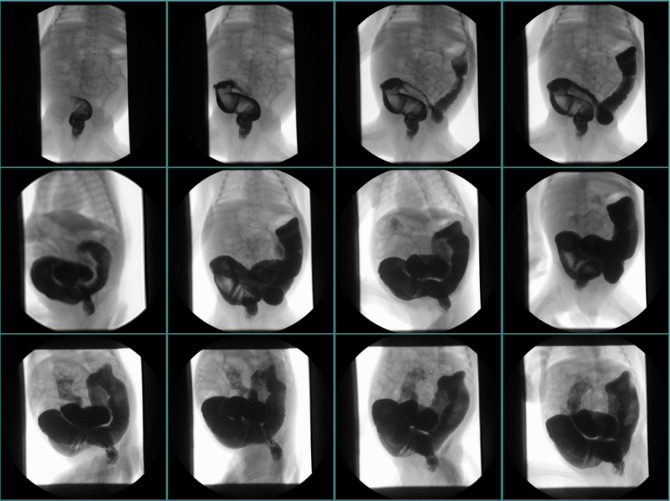
Pre- and postoperative irrigography

**Figure 2:**
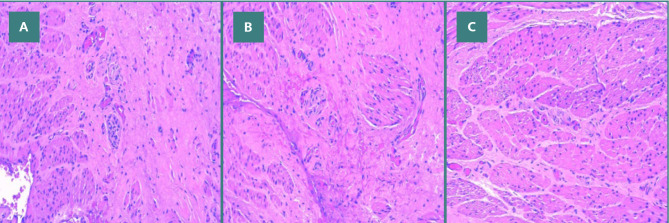
Rectal biopsy specimen showing hyperplasia of neural filaments in the myenteric plexus, with no ganglion cells identified on hematoxylin and eosin stain. A, Hyperplasia of neural filaments; B, Aganglionosis in the myenteric plexus; C, Hyperplasia of neural filaments and aganglionosis in the myenteric plexus on hematoxylin and eosin stain.

**Figure 3:**
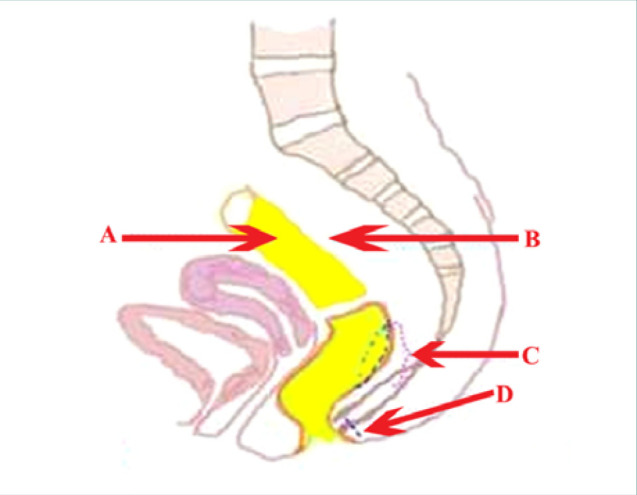
Schematic representation of aganglionic segment resection and sigmoid colon mobilization during Duhamel procedure. A, Aganglionic segment to be resected; B, Region of the sigmoid colon with aganglionosis; C, Proximal colostomy segment mobilized toward the posterior rectum, the transanal excision performed having a diameter at least two times greater than the diameter of the descended intestine; D, Lateral anal sphincterotomy with sphincter and anal opening weakening. Source: Handaya AY, Fauzi AR, Andrew J, Hanif AS. Modified Duhamel with lateral anal sphincterotomy and coloanal stump for adult Hirschsprung's disease: A case series. Int J Surg Case Rep. 2020;77:174-177. doi: 10.1016/j.ijscr.2020.10.066.

**Figure 4:**
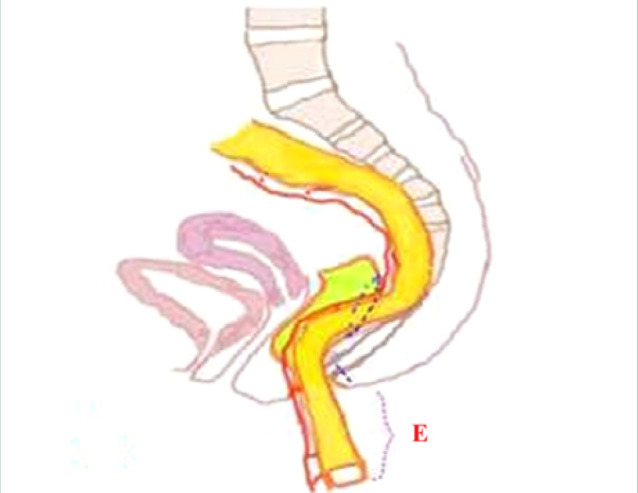
Visualization of intestine lowering and mobilization in relation to the anal canal following surgical intervention. E, Intestine lowered and mobilized at least 10 cm outside the anal canal. Source: Handaya AY, Fauzi AR, Andrew J, Hanif AS. Modified Duhamel with lateral anal sphincterotomy and coloanal stump for adult Hirschsprung's disease: A case series. Int J Surg Case Rep. 2020;77:174-177. doi: 10.1016/j.ijscr.2020.10.066.

Figures 1-4 present irigographies, rectal biopsy and schematic representation of the surgery.

Microscopically, the paraffin-embedded histopathological examination of the biopsy from the intestinal muscle wall (excluding mucosa and submucosa) revealed rare neurofilaments, with no ganglion cells observed in the sampled fragment (Figure 2 A-C).

After 2 years, the patient, known to be suffering from congenital megacolon and with a right-side colostomy that was performed at the age of 5 months, with constant evolution and increasing weight, returned to the hospital for specialized treatment. A Duhamel-type extramucosal sphincter-rectal-myotomy was performed with favorable evolution [7,8].

**Figure 5:**
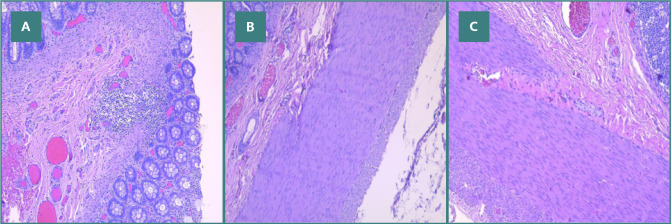
Colectomy specimen with muscular wall present on 5.5 cm proximally with a maximum caliber of 1 cm. A, Submucosa with hyperplasia of neural filaments with a filiform appearance; B, Colonic wall with autonomic innervation in the submucosa or muscularis propria; C, Colonic wall with aganglionosis of the submucosal and myenteric plexuses.

**Case 2:** A 1-year and 6-month-old patient with a history of congenital megacolon, who had undergone colostomy and ileostomy at 2 months of age, presented for further surgical treatment. The patient had shown constant clinical progression. A specimen of rectal mucosa and submucosa was sent for paraffin histopathological examination. The biopsy fragment collected from the colonic mucosa showed a preserved glandular architecture, hypertrophic muscularis mucosae with a small area of edematous submucosa with rare neurofilaments and no ganglion cells on the examined sections (Figure 5 A-C).

**Figure 6:**
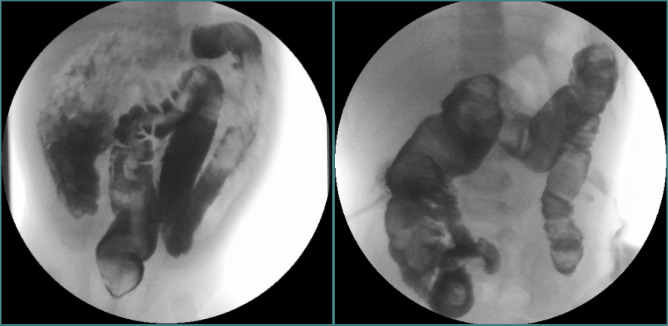
Pre- and postoperative irrigography

Microscopic examination revealed aganglionosis in the rectum and sigmoid colon segments, as well as neurofilament hyperplasia and fibrosis in the submucosa. The vegetative innervation appeared preserved but reduced and unevenly distributed in the descending, transverse, and ascending colon. Additionally, granulomatous inflammation characterized by a foreign-body giant cell reaction was observed in the serosa [9,10]. The anatomical pathology diagnosis indicated that the histopathological features were consistent with Hirschsprung’s disease affecting the rectum and sigmoid. This condition is associated with hypoganglionosis extending through the descending, transverse, and ascending colon. Pre- and postoperative irrigography revealed a nearly normal rectosigmoid index—dolichosigmoid with decalibration in the middle portion of the transverse colon. The ascending and transverse colon exhibited hypokinesis with an obviously enlarged ahaustral caliber, while the descending colon maintained a quasi-normal caliber (Figure 6) [6,11].

**Figure 7:**
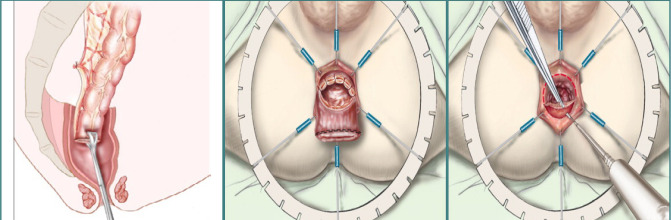
The Soave procedure. Source: Valverde A, Goasguen N, Oberlin O, Mosnier H. Video-assisted transanal proctectomy using the SILS® monotrocar. J Visc Surg. 2013 Feb;150(1):33-43. doi: 10.1016/j.jviscsurg.2013.01.008

A Soave-type endoanal descent and ileostomy closure were performed, resulting in a favorable postoperative outcome. The Soave procedure involves the descent of the normal colon, which is innervated by a sleeve of rectal muscles, into the perianal position (Figure 7). This technique preserves the pelvic vasculature and innervation while maintaining the integrity of the internal sphincter. Despite potential concerns, an increase in postoperative constipation has not been clinically confirmed [11,12].

## CONCLUSION

Prompt diagnosis and appropriate surgical management are critical in the treatment of Hirschsprung’s disease. Modern surgical techniques, such as the Duhamel sphincter-rectal-myotomy and the Soave endorectal pull-through, are instrumental in restoring intestinal function and significantly enhancing the quality of life for affected patients. Effective postoperative care is essential to prevent complications and ensure optimal recovery, underscoring the need for specialized postoperative management. These treatments aim to reestablish intestinal continuity and restore normal colon function, ultimately improving patient outcomes.
